# New insights on thyroid hormone mediated regulation of herpesvirus infections

**DOI:** 10.1186/s13578-017-0140-z

**Published:** 2017-03-21

**Authors:** Robert W. Figliozzi, Feng Chen, S. Victor Hsia

**Affiliations:** 1 Department of Pharmaceutical Sciences, School of Pharmacy and Health Professions, University of Maryland Eastern Shore, Princess Anne, USA; 2 Department of Natural Sciences, School of Agriculture and Natural Sciences, University of Maryland Eastern Shore, Princess Anne, USA

**Keywords:** Herpes simplex virus, Thyroid hormone, Differentiation, Casein kinase 2, Phosphoinositide 3-kinase, Tetrabrominated cinnamic acid, 4,5,6,7-Tetrabromo-2H-benzotriazole, 2-Morpholin-4-yl-8-phenylchromen-4-one

## Abstract

Thyroid hormone (T_3_) has been suggested to participate in the regulation of herpesvirus replication during reactivation. Clinical observations and in vivo experiments suggest that T_3_ are involved in the suppression of herpes virus replication. In vitro, differentiated LNCaP cells, a human neuron-like cells, further resisted HSV-1 replication upon addition of T_3_. Previous studies indicate that T_3_ controlled the expression of several key viral genes via its nuclear receptors in differentiated LNCaP cells. Additional observation showed that differentiated LNCaP cells have active PI3K signaling and inhibitor LY294002 can reverse T_3_-mediated repression of viral replication. Active PI3K signaling has been linked to HSV-1 latency in neurons. The hypothesis is that, in addition to repressing viral gene transcription at the nuclear level, T_3_ may influence PI3K signaling to control HSV-1 replication in human neuron-like cells. We review the genomic and non-genomic regulatory roles of T_3_ by examining the phosphoinositide 3-kinase (PI3K) pathway gene expression profile changes in differentiated LNCaP cells under the influence of hormone. The results indicated that 15 genes were down-regulated and 22 genes were up-regulated in T_3_-treated differentiated LNCaP cells in comparison to undifferentiated state. Of all these genes, casein kinase 2 (CK2), a key component to enhance PI3K signaling pathway, was significantly increased upon T_3_ treatment only while the cells were differentiated. Further studies revealed that CK2 inhibitors tetrabrominated cinnamic acid (TBCA) and 4, 5, 6, 7-tetrabromo-2H-benzotriazole (TBB) both reversed the T_3_-mediated repression of viral replication. Together these observations suggested a new approach to understanding the roles of T_3_ in the complicated regulation of HSV-1 replication during latency and reactivation.

**Electronic supplementary material:**

The online version of this article (doi:10.1186/s13578-017-0140-z) contains supplementary material, which is available to authorized users.

## Background

The herpes viruses, herpes simplex 1 (HSV-1) and herpes simplex 2 (HSV-2) are infamous to the general public for causing unsightly and painful oral and genital lesions [1]. Curiously the third member of the alpha human herpes virus family (αHHV), human herpes virus 3, or varicella zoster virus (VZV), commonly known as the chicken pox or shingles virus, is considered less of a taboo. This is perhaps due to the success and ubiquity of the VZV vaccine in the late 1980s and that lesions from VZV rarely present themselves more than a few times in a patient’s life, usually during early childhood and late adulthood [2]. Conversely, HSV-1 and HSV-2 symptoms occur sporadically throughout the patient’s lifetime with little predictability. It is this alternating duality between symptomatic, lytic, and asymptomatic, latent, periods that led to the name herpes or creeping from Latin. In addition to having lytic and latent periods, these herpes viruses have similar virion structures, protein functionality, genetic similarity, cause epithelial lesions and the affinity to reside almost exclusively in sensory ganglion during latency. Ironically the biological mechanisms that determine when and how these viruses exit latency and produce lytic symptoms is still undefined. Researchers believe that a complex relationship between the host’s immune system, nervous system, infected cell signal transduction, infected cell transcriptional regulation, and stress from the host’s environment is responsible for the switch.

Interestingly thyroid hormones, play roles within the immune system, nervous system, cell signal transduction, transcriptional regulation, etc. and T_3_ fluctuations are often linked to environmental stress [1]. These associations led to the hypothesis that thyroid hormones play a role in the suppression and reactivation of herpes viruses. To test this hypothesis our lab has studied the effect of thyroid hormone treatments on HSV-1 infections using different models. In addition, our lab has reported results from two retrospective clinical data analyses where patients with thyroid hormone complications increased the odds ratio of having herpes virus reactivation [3, 4]. The first study identified that several specific age/gender hospitalized patient groups at a comprehensive research medical center in urban Taiwan, with thyroid disorders were 2 times more likely to also have a αHHV [4]. The second study identified that hospitalized patients at a regional hospital in rural Maryland with thyroid disorders were 3 times more likely to have VZV diagnoses [3]. To understand these clinical observations, our lab studies cellular thyroid hormone action in regards to transcriptional regulation and signal transduction and have found that both mechanisms might be affecting HSV-1 infections.

The nuclear activity of T_3_ and its receptor (TR) family has been studied for decades [5–22]. The most well characterized mechanisms involve the transcriptional regulation of genes that are transcriptionally repressed in the absence of T_3_ and activated upon ligand TR. Most these genes contain a T_3_ response element (TRE) within its promoter. The traditional TRE, known as a direct repeat 4 (DR4) is characterized by containing two hexameric half-sites, with a 5′-AGGTCA-3′ consensus sequence, separated by any 4 nucleotides. Typically, the TR DNA binding domain (DBD) binds to the downstream half-site with retinoic acid X receptor (RXR) occupying the upstream half-site, forming a heterodimer. TR homodimers are also reported. In the absence of T_3_, the complex either bind loosely allowing repressive histones to block transcription or the complex can participate in recruiting repressive histone modifying enzymes. Upon T_3_ binding to the TR the complex undergoes a conformational change which recruits transcription activating histone modifying enzymes. Other, less common, TRE arrangements such as single half-sites, inverted repeats (IR) and palindromes found on the TSHβ, lysozyme silencer, and TSHα genes (respectively) are not as well characterized. Epidermal growth factor receptor, myosin heavy chain β, prolactin, thyroid-stimulating hormone α, thyroid-stimulating hormone β, thyrotropin-releasing hormone, type II 5′-deiodinase and the HSV-1 TK promoter and impart a regulatory pattern seemingly opposite of the traditional DR4 TREspositive regulation [22–26]. When T_3_ is absent genes with these negative TRE (nTRE) are transcriptionally activated and upon T_3_ binding the transcription is repressed. These nTREs are found on the promoters of genes that well known to be repressed by T_3_ feedback inhibition.

T_3_ was also shown to influence PI3K signaling [27, 28]. In addition, the hormone exhibited non-genomic functions to control physiological functions. The actions were initiated by receptors at the plasma membrane or in the cytoplasm. The receptors mentioned in this category are either TR isoforms or integrin, for example, αvβ3 [29]. For example, TR is reported to interact with the Pi3K regulatory subunit Pi3KR1 resulting in increased Pi3K activity. Therefore, it appeared that T_3_/TR used many mechanisms to expand their regulatory roles in biology. However, it is still unclear regarding its molecular mechanisms.

Differentiated human LNCaP cells have been developed as a proxy of neurons for investigating the regulation of HSV-1 gene expression and replication [30–32]. This differentiated cell line is not a true sensory neuron of trigeminal ganglia or dorsal root ganglia where HSV-1 usually infected during latency, but demonstrated important human neuron-like morphology and physiology. The cells following differentiation exhibited long neurite-like processes, rounding of the cell body, the presence of secretory granules as well as physiological markers such as the expression of chromogranin-A, differentiation-specific ionic conductances, neuron-specific enolase (NSE), and the secretion of mitogenic neuropeptides neurotensin, and parathyroid hormone-related peptide [33–36].

### Herpes virus transcriptional regulation by TR and T_3_

Several decades old and our recent studies have explored the nTRE in the promoter of the HSV-1 thymidine kinase (TK) gene [37–39]. Initially this promoter was believed to be insensitive to treatments in most cells but activated in pituitary cells upon T_3_ [39]. More recently it has been shown that T_3_ can cause the repression of TK transcription in the certain neuron-like differentiated cell types that expresses the appropriate cofactors [32, 37]. These conditions mirror the only cellular environment where herpes virus latency exists, sensory neurons. In addition, our lab showed that T_3_ treatment of these infected differentiated neuron-like cells had markedly reduced HSV-1 replication compared to controls. The virus retained the ability to replicate normally after the T_3_ was removed from the system, mimicking latency and reactivation [32, 40]. Our observations however, puzzle our virologist colleagues since HSV-1 TK is not considered an essential gene to viral replication. Therefore, we continue to explore other mechanisms that support our findings. In a parallel we have tested the ability of T_3_ to repress the VZV nucleotide kinase (VZV-PK) in transfection experiments. Similarly, to transfection experiments with HSV-1 TK, VZV-PK promoter activity is also repressed by T_3_ treatment [3].

### T_3_ signal transduction regulation

It has been realized that signaling pathway activated via PI3-kinase (PI3K) and Akt is necessary to repress HSV-1 reactivation [41]. Studies indicated that PI3K activation by nerve growth factor (NGF) interaction with its high-affinity tropomyosin receptor kinase (TrkA) generated a cascade of signals resulting in neuronal gene expression changes thus promoting latent infection. This observation was supported by a number of investigations showing that addition of anti-NGF antibodies to the explanted trigeminal ganglia (TG), superior cervical ganglia **(**SCG), and eyes of latently infected animals causing more virus shedding and increased reactivation  [42]. Several downstream targets of PI3K/Akt pathway were discussed in terms of their functions in latency and reactivation. For instance, the mTORC1 kinase is one of the primary objects and it played a critical role in maintaining latency  [43]. The mTORC1 was sufficient to regulate many proteins including eIF4E-binding proteins (4E-BPs), which is a host cell translation repressor controlling cap-dependent mRNA translation and temporary disruption was sufficient to reactivate the virus [43]. Factors/episodes participating in altering PI3K/Akt pathway may have a role in modulating HSV-1 latency and reactivation but the detailed mechanisms were unclear.

Previous reports showed that without affecting cell viability T_3_ was sufficient to control HSV-1 gene expression and replication in human neuron-like cells by targeting key viral genes [1, 30–32, 37]. It is not known if the hormone influenced the PI3K/Akt cascaded to produce the regulation. Our ongoing study attempts to investigate the gene expression profile changes upon T_3_ treatment comparing differentiated and undifferentiated conditions. Several genes exhibited significant expression level changes, and function inhibition of one gene reversed T_3_-mediated repression and promoted viral replication.

### HSV-1 infected murine trigeminal ganglion (TG) explant

**Fig. 1:**
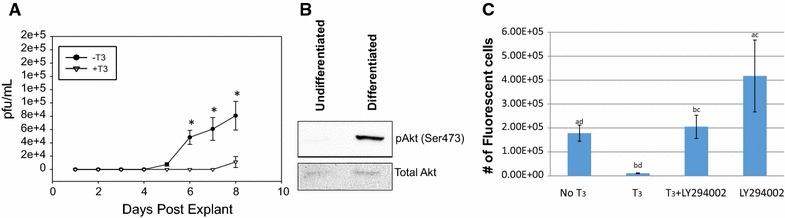
**A** HSV-1 infectious viral particles (ivp) released from T_3_ treated latently infected mouse TG explants. TGs from mice n = 10 latently infected with HSV-1 were explanted 30 days post infection. TG explants were separated into replicates of two treatment groups, +T_3_ and −T_3_, and were cultured for 8 days post explant. Media from each replicate was quantitatively tested daily for HSV-1 ivp via plaque assay. Two-way ANOVA with Holm-Sidak post hoc analysis suggests that statistically significant differences in ivp between +T_3_ and −T_3_ treatment at days 6, 7, and 8. *Asterisk* denotes p < 0.001. **B** PI3K Pathway is active in differentiated LNCaP cells with pAkt increase in differentiated cells. Western blot was performed using rabbit monoclonal IgG antibody against phospho-AKT pSer473 (ThermoSci, Cat#: OMA-03061) and mouse antibody AKT (Rockland, Cat#: 200-301-401) at a dilution of 1:1000 followed by the addition of conjugated secondary antibody for detection on extract from undifferentiated and differentiated LNCaP cells. **C** PI3K inhibitor reversed T_3_-mediated repression HSV-1 viral replication from differentiated LNCaP cells treated with 100 nM T_3_ and/or 20 µM LY294002 (Sigma Aldrich, cat#: L9908) was measured quantitatively by FLICIT assays [68]. In short, Vero cells were seeded on 384-well plates followed by exposure to media from EGFP HSV-1 infected cultures. The infected media samples were applied in serial dilutions in replicates and were incubated for 8–18 h when EGFP was observed. The numbers of total cells and infected cells were imaged and quantified by the BioTek Cytation3 fluorescent imaging station and Gen5 software then used to calculate the viral titer using an inverse Poisson’s equation as previously described. Two-way ANOVA with Holm-Sidak post hoc analysis suggests that statistically significant differences in fluorescently labeled infected cells per mL exists. *a* p < 0.018, *b* p < 0.004, *c* p < 0.012, *d* p < 0.035

To correlate our clinical findings with our molecular biology data and our hypothesis we performed a small animal experiment. Explanted TG from mice latently infected with HSV-1 treated with T_3_ exhibited delayed viral release compared to no treatment (Fig. 1A). Over the 8-day period post explant, samples from the two culture groups were analyzed by plaque assay for infectious viral particles (ivp). The untreated group began releasing measurable ivp at day 5 which increased over the remaining days of the experiment. The T_3_ treated sample did not release measurable particles until the 8th day, which were fourfold lower compared to the untreated explants (Fig. 1A).

### PI3K/Akt pathway is active in differentiated LNCaP cells and contributed to T_3_-mediated regulation of HSV-1 replication

Differentiated LNCaP cell is considered a human neuron-like cells due to its physiological similarity to neurons. We have developed a protocol (T_3_ removal assays) to measure the effects of hormone on neurotropic virus replication such as HSV-1 [32, 40]. In short, two groups of cells were infected under T_3_ for 48 h then the hormone was removed from one group and the T_3_ regulatory effects were measured by either plaque assays or FLICIT assays at 96 hpi [44]. It was speculated that PI3K/Akt signaling is active in differentiated LNCaP since it was very suppressive to HSV-1 replication in comparison to undifferentiated condition [32, 40]. This hypothesis was tested first by Western blot analyses using antibodies against total Akt and phospho Akt (pAkt) on extracts from undifferentiated and differentiated LNCaP cells. The results demonstrated that the level of pAkt was quite low if there was any in undifferentiated cells but significantly increased when cells were differentiated (Fig. 1B). The PI3K suppressive effects on HSV-1 replication was studied by inhibitor LY294002, which was shown to reactivate HSV-1 from latency by blocking PI3K pathway [41, 43, 45]. The results showed that LY294002 reversed T_3_-mediated repression (Fig. 1C). These observations together indicated that differentiation activated the PI3K/Akt signaling pathway of LNCaP cells and this activation participated in the T_3_-mediated repression of HSV-1 replication.

### PI3K pathway related gene expression profiles of differentiated LNCaP under T_3_ treatment

**Fig. 2:**
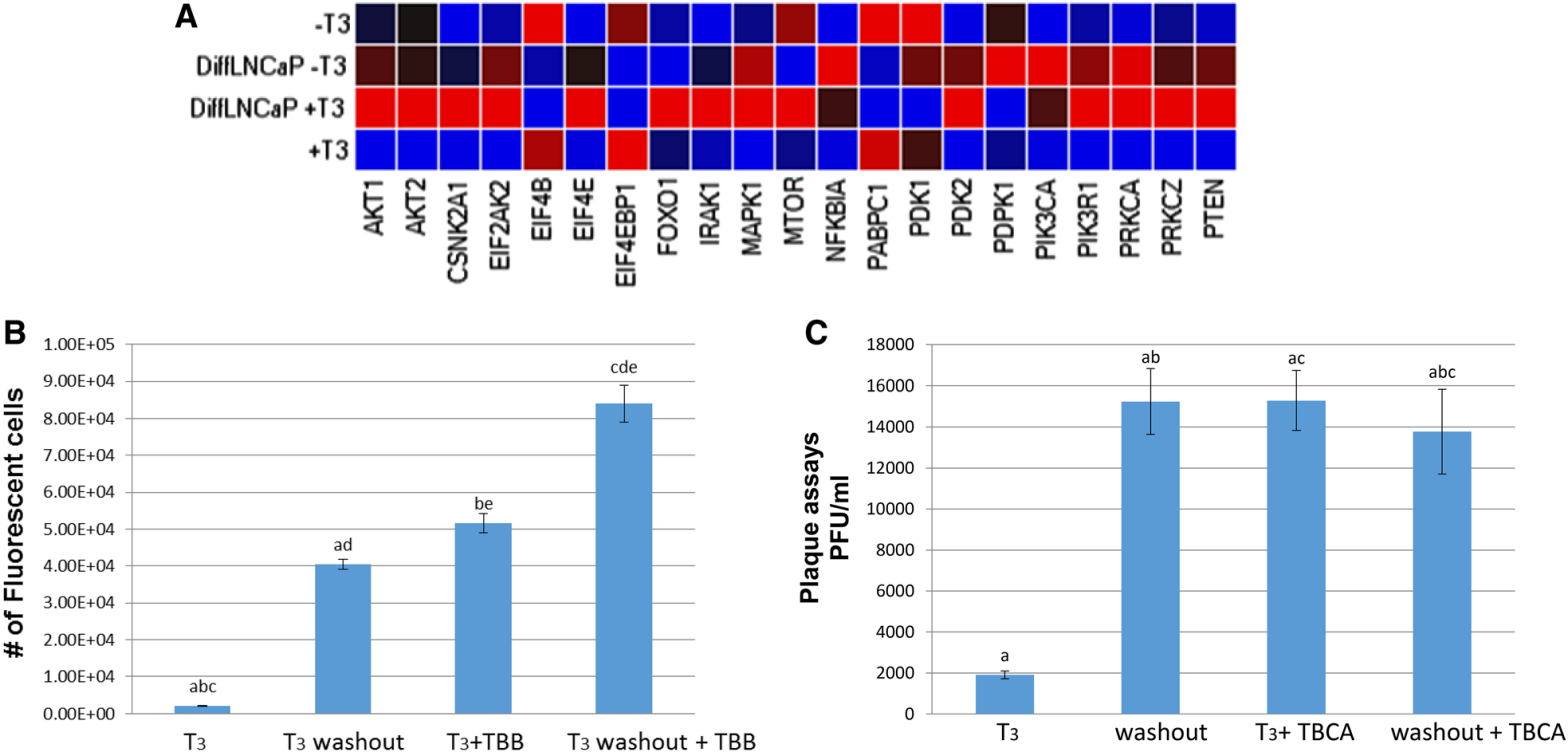
**A** Transcription profiles of genes involved in PI3K/Akt pathway measured by qRT-PCR arrays. Undifferentiated and 5-day differentiated LNCaP cells plated on poly-d-lysine coated T75 flasks were treated with and without 100 nM T_3_ for 48 h. The total RNA was purified by TRIZOL and the cDNA was synthesized using RT2 first strand kit (QIAGEN, cat#: 330401). For the transcriptome heatmaps assessment, the cDNA was subjected to qRT-PCR array analyses via PI3K-AKT signaling pathway (SAB Target List) H96 (BIO-RAD, cat#: 100-34223). The protocol was described essentially by the manufactures based on the CFX Connect™ Real-Time PCR Detection System (BIO-RAD Cat# 1855200). Amplification was plotted and analyzed in triplicates using the BIO-RAD CFX manager software provided by the manufacturer. For each gene, the brightest* red squares* indicate at least a fourfold increase over the brightest* blue square*.** A** Showed the select genes from PI3K-AKT target list modulated significantly by T_3_ treatment and differentiation. Akt, EIF, and mTOR genes regulated by T_3_ and differentiation. **B** CK2 inhibitor TBB disrupts T_3_ mediated reduction of viral replication. The viral replication was measured by T_3_ removal assays [32] and FLICIT assays as shown in** B** with modification. TBB (Santa Cruz Bio, cat#: sc-202830) was added at 1 µM for CK2 inhibition. In short, differentiated cells were infected with HSV-1. At 48 hpi, infected cells were treated with (1) T_3_, (2) T_3_ washout, (3) T_3_ plus TBB, or (4) T_3_ washout plus TBB. The culture media were collected at 96 hpi and subjected to PLICIT assays. The results showed that infection with 100 nM of T_3_ reduced viral replication and hormone washout reversed this reduction. The addition of TBB disrupted the T_3_–mediated repression. FLICIT was reported previously [68] and described in the figure. Data in triplicates, two-way ANOVA with Holm-Sidak post hoc analysis suggests that statistically significant differences in fluorescently labeled infected cells exists; *a*, *b*, *c*, *d*, *e* p < 0.001. **C** TBCA reversed T_3_-mediated suppression of viral replication in differentiated cells. LNCaP cells were infected under treatment of no T_3_, with T_3_, 110 nM TBCA (Millipore, cat#: 218710), or T_3_ + TBCA followed by plaque assays to measure the release of infectious viruses. No suppression of viral replication was observed for undifferentiated cells under the influence of T_3_ and/or TBCA when analyzed by ANOVA (data not shown). T_3_ removal assays as described in **A** were used to investigate the effects of TBCA. At 48 hpi, infected cells were treated with (1) T_3_, (2) T_3_ washout, (3) T_3_ plus TBCA, or (4) T_3_ washout plus TBCA. It is shown that TBCA, similar to TBB, reversed the suppression of viral replication by T_3_ as measured by viral plaque assay. Data in triplicates were analyzed by Two-way ANOVA with Holm-Sidak post hoc analysis suggests that statistically significant differences in pfu per mL exists; *a* p < 0.001, *b* p < 0.046, *c* p < 0.040

To address the impact of T_3_ on the PI3K pathway in differentiated cells compared to undifferentiated conditions, we performed quantitative PrimePCR^®^ PI3K-Akt Array Assays to measure the expression profile of PI3K related genes. 84 genes were analyzed (complete data in Additional file 1: Figure S1). Of all these genes, the expression of 15 genes were decreased and 22 genes were increased significantly in T_3_-treated differentiated LNCaP cells when compared to undifferentiated LNCaP (Fig. 2A). For example, eIF4E and its regulator eIF4EBP1 showed opposite expression profile (Fig. 2A). To be specific, eIF4E from differentiated cells was identified to have sevenfold expression increase in comparison to undifferentiated condition. eIF4EBP1, however, exhibited fivefold decrease. In addition, eIF2AK2, commonly known as PKR, reported to play a role in blocking HSV-1 translation, displayed a two-fold increase in T_3_-treated differentiated cells (Fig. 2A). Together the analyses suggested that PI3K gene expression was hugely influenced by T_3_ and may have critical roles in controlling viral replication in differentiated condition.

### Roles of casein kinase 2 in HSV-1 replication in T_3_-treated differentiated LNCaP cells

Casein kinase 2 (CK2) is a serine/threonine protein kinase that targets a number of proteins such as casein [46]. The kinase is composed of a tetramer of α, α’, and two β subunits [47, 48]. The PrimePCR assays showed that casein kinase 2 α1 (CSNK2A1) was significantly upregulated in T_3_-treated differentiated LNCaP cells (Fig. 2A). It was shown that CK2 can promote PI3K/Akt signaling by inhibiting PTEN, a suppressor of Akt/PKB signaling pathway [49–54]. To test the hypothesis that TH promoted HSV-1 replication suppression in differentiated LNCaP by enhancing PI3K signaling via CK2, an inhibitor of CK2, TBB was first used in HSV-1 infection of differentiated LNCaP cells in the presence of T_3_. The results indicated that hormone was suppressive to the viral replication and removal of T_3_ at 96 hpi activated the viral replication repressed by T_3_, suggesting the experiment was valid (Fig. 2B). The TBB treatment somehow overturned the T_3_-mediated suppression (Fig. 2B). It is likely due to the blocking of the CK2 activity.

Although TBB is widely used as a CK2 inhibitor, it was reported to have more effects on other kinase [55–58]. To confirm the roles of CK2 in this T_3_-mediated HSV-1 replication regulation, a recently reported CK2 inhibitor, TBCA, was used since it exhibited more specific inhibition on CK2 [59, 60]. To distinguish the importance of differentiation, undifferentiated cells were infected in the presence of T_3_ with or without TBCA and the results demonstrated that there was no difference in terms of the strength of viral replication (data not shown). However, when the cells were differentiated, T_3_ repressed the viral replication and hormone washout at 96 hpi recovered the viral replication previously blocked by T_3_ (Fig. 2C). The TBCA treatment, like the TBB, abolished the T_3_-mediated suppression (Fig. 2C). Together these results supported the hypothesis that increased expression of CK2 by T_3_ may have a role in modulating PI3k/Akt pathway in differentiated human neuron-like cells to suppress HSV-1 replication.

## Conclusions

Using this model, we were able to address the importance of differentiation during HSV-1 latency since the HSV-1 infection of undifferentiated LNCaP was very efficient and the differentiation significantly decreased the viral replication [31, 32, 40]. However, it is important to realize the limitations of this model. For example, it is a human neuroendocrine prostate cancer cell line and can only serve as an in vitro model without reflecting the real situations of latent infections. HSV-1 replication, although reduced dramatically, never established a *bona fide* latency in this model.

With these limitations in mind, this model has several advantages for HSV-1 study. First, it can be easily induced to differentiate simply by androgen deprivation [61] with consistent results and the differentiation is usually achieved within 2 weeks and the cells can survive in this condition for up to a month with normal culture condition without the addition of NGF. In addition, these infected cells when treated with T_3_ exhibit a marked reduction in HSV-1 replication and release. While not considered a gene essential to replication in lytic infections, HSV-1 TK transcription is substantially reduced upon T_3_ treatment [62]. TK has been referenced as one of the necessary genes for efficient reactivation in neurons since other genes are also expressed at the very beginning of the reactivation [63, 64]. This leads us to consider the transcriptional regulation of TK by T_3_, one of several factors in the control and switch between herpes latency and reactivation. We further hypothesize that other additional T_3_ mechanisms, such as PI3K signaling, also play a role in this complex switch.

While cytoplasmic TR acting with PI3K has been reported we have not yet explored this mechanism experimentally in our system. We plan to further investigate the roles of both genomic and nongenomic TR action using siRNA against key Pi3K, CK2, and TR subunits and isoforms. Currently our data supports that T_3_/TR viral suppression is due to genomic suppression of the viral genome and genomic regulation of CK2 and Pi3K pathway components which leads to additional nongenomic regulation. Furthermore, we have identified putative TREs on the promoter region of CK2 and plan to confirm them with a series of mutation experiments and electromobility shift assays.

The relationship between T_3_ and CK2 was not extensively investigated. Most studies showed that CK2 phosphorylated TR isoforms or corepressor [20, 65, 66]. Thyroid hormone was reported to enhance casein kinase activity in the liver of rat [67]. In our study, HSV-1 replication was retarded and inhibitors against CK2 was sufficient to rescue the virus’s ability to replicate at normal levels. Based on our clinical, in vivo, in vitro, and molecular biology observations, it is likely that both genomic and non-genomic effects of T_3_ play a role in the suppression of herpesvirus infection and potentially participate in the complex regulation of latency and reactivation.

## Additional file

**Additional file 1: Figure S1.** BIO-RAD PrimePCR, qRT-PCR array, human PI3K-AKT signaling pathway (SAB target list) H96. Complete 84 gene expression heatmap. See main text for assay details.

## Abbreviations

HSV-1: herpes simplex virus type-1
T_3_: thyroid hormone
TBCA: tetrabrominated cinnamic acid
TBB: 4,5,6,7-tetrabromo-2H-benzotriazole
LY294002: 2-morpholin-4-yl-8-phenylchromen-4-one
hpi: hours post infection
FLICIT: fluorescently labeled infected cell inoculum titration
moi: multiplicity of infection
