# Supplementary material for: New insights on thyroid hormone mediated regulation of herpesvirus infections
Source: Cell Biosci. 2017 Mar 21;7:13. doi: 10.1186/s13578-017-0140-z (PMC5360088; doi:10.1186/s13578-017-0140-z)
Supplement: Supplementary file 1 — Additional file 1: Figure S1. BIO-RAD PrimePCR, qRT-PCR array, human PI3K-AKT signaling pathway (SAB target list) H96. Complete 84 gene expression heatmap. See main text for assay details. [file 13578_2017_140_MOESM1_ESM.pdf]

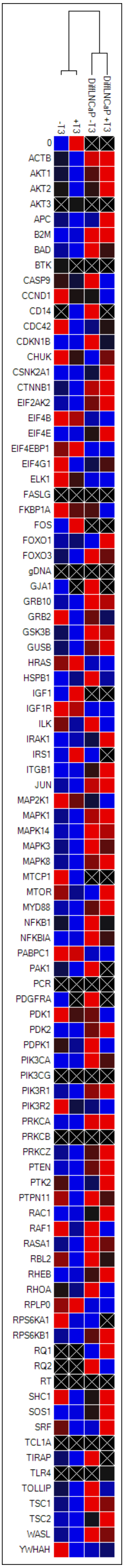

Figure S1. Bio-Rad PrimePCR, qRT-PCR Array, Human PI3K-AKT signaling pathway (SAB Target List) H96. Complete 84 gene expression heatmap. See main text for assay details.
